# Mechanical Analysis of 3D Printed Polyamide Composites under Different Filler Loadings

**DOI:** 10.3390/polym15081846

**Published:** 2023-04-11

**Authors:** Nabilah Afiqah Mohd Radzuan, Nisa Naima Khalid, Farhana Mohd Foudzi, Nishata Royan Rajendran Royan, Abu Bakar Sulong

**Affiliations:** 1 Department of Mechanical & Manufacturing Engineering, Faculty Engineering & Built Environment, Universiti Kebangsaan Malaysia, Bangi 43600, Selangor, Malaysia; 2 Fuel Cell Institute, Universiti Kebangsaan Malaysia, Bangi 43600, Selangor, Malaysia; 3 School of Engineering, UOW Malaysia KDU University College, Glenmarie Campus, Shah Alam 40150, Selangor, Malaysia

**Keywords:** polymer composites, fused deposited modelling, layup sequence, mechanical properties

## Abstract

The production of fabricated filaments for fused deposited modelling printing is critical, especially when higher loading filler (>20 wt.%) is involved. At higher loadings, printed samples tend to experience delamination, poor adhesion or even warping, causing their mechanical performance to deteriorate considerably. Hence, this study highlights the behaviour of the mechanical properties of printed polyamide-reinforced carbon fibre at a maximum of 40 wt.%, which can be improved via a post-drying process. The 20 wt.% samples also demonstrate improvements of 500% and 50% in impact strength and shear strength performance, respectively. These excellent performance levels are attributed to the maximum layup sequence during the printing process, which reduces the fibre breakage. Consequently, this enables better adhesion between layers and, ultimately, stronger samples.

## 1. Introduction

Fused deposited modelling (FDM) appears to be a promising technology to employ in 2023, as research in this area has advanced since open-source printers became available on the market. Previous studies have indicated that having successfully printed composites may bring about industrial advantages due to their ability to broaden the economic impact whilst increasing the recyclability of polymeric composite materials [1]. Achieving a proper understanding and functional capability of polymeric composite samples or products through FDM printing will allow greater versatility, especially in metal–composite materials [2]. Open-source printers can usually utilise materials from any source, which has encouraged researchers to explore this area more extensively, especially in terms of filler loading. Theoretically, as the filler loading increases, the mechanical properties improve, as reported in a study conducted in the 1990s [3]. In 2022, a study on continuous fibre composites (45 vol.%) recommended controlling fibre as an inner structure of the filament while maintaining the unidirectional fibre orientations at optimum performance levels (27 GPa) [4]. These are potentially promising techniques to adopt, especially when applied to metal–composite materials [5]. Previous studies on polymeric composites have reported that at higher glass fibre loadings (40 wt.%), the composites experience deterioration (27%) in terms of the Young’s modulus [6]. Research has clearly indicated that, although obtaining higher loadings will boost the mechanical performance, having these materials in filament form (1.75 mm diameter) will not ensure the enhancement of mechanical properties [7,8]. The rheological properties and mechanical behaviour of materials with varying filler loadings have frequently been examined in previous studies [9]. However, limited research has been undertaken on materials in the form of filaments, despite this being a promising area for future development. The BASF–SE company introduced polymer–metal composites in filament form with up to 80 wt.% of stainless steel 316 L, indicating that even higher loading fillers could be printed using FDM techniques. Therefore, the effects of different filament loadings on the mechanical performance must be better understood as part of the advance towards 4D printing.

Theoretically, reinforcing fillers/fibres with polymeric materials can increase the mechanical performance of composite materials. However, the composition of each fibre is subject to the fabrication process. Studies on nylon-reinforced carbon, glass, and Kevlar materials indicated that the maximum tensile strength is 524 MPa [10]. Meanwhile, other studies conducted using short carbon fibre reinforced polyamide indicated a maximum of 2.4 to 2.6 GPa [11]. Based on this research, it is unclear how much filler or added fibre is needed to enhance the mechanical performance. To date, conventional fabrication processes, including injection or compression moulding, still have limitations in terms of the filler compositions used. Studies on compression techniques have indicated that a maximum filler addition of ~10 wt.% is required to improve mechanical properties before the results plateau or deteriorate [12,13]. Therefore, having a detailed understanding of the maximum filler additions on mechanical performance is crucial, especially when fused deposited modelling techniques are involved. Additionally, studies on polymeric composite filaments suggest that controlling the particle size in the range of 8–25 nm enables shear thickening at high shear rates [14]. However, the layer thickness needs to remain below 20–30 nm to increase the polymer absorption on solid surfaces. This phenomenon occurs because the polymer chain can extend to become a more bridging structure. Meanwhile, another study recommended the use of the woven printing technique to reduce filler breakage, minimise the printing void, and increase adhesion between the printed layers [15,16,17]. Nevertheless, this would further contribute to shear stress, which is influenced by the use of different printing techniques, printing behaviours, or even filler sizes during the filament fabrication process [18]. One study revealed that the shear stress on a printed filament must be considered, as moderate shear stress would allow a better distribution, while the highest level would cause the printed layer to be dispatched [19]. Printed layers are crucial, as they allow load to be transferred between the adjacent layers, thus minimising the critical failure [20]. Moreover, rather than having adjacent printed layers, having suitable geometrical filler shapes is also crucial. One study indicated that fully continuous fibres with a higher shear strength (40.9 MPa) are superior fillers than short carbon fibres (24.4 MPa) [20,21]. Therefore, in this study, we incorporated an adjacent layup sequence and filler loadings to ensure strong adhesion occurred between the printed layers of polyamide reinforced carbon fibre. This study also investigated the difference between conventional filaments (which are prescribed by companies) and filaments prepared in-house with similar compositions. 

## 2. Experimental Method

### 2.1. Materials and PACF Preparations

**Figure 1:**
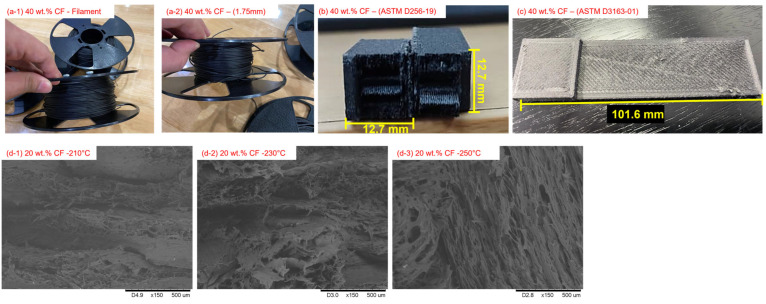
(**a-1**) Images of the 40 wt.% filament (**a-2**) with a 1.75 mm diameter fabricated using the 3Devo filament maker; (**b**) 40 wt.% impact test samples after breakage (cross-section view); (**c**) one-sided shear test sample with a composition of 40 wt.% CF; and micrograph images of printed 20 wt.% samples at several temperatures: (**d-1**) 210 °C, (**d-2**) 230 °C and (**d-3**) 250 °C.

The main filler used for these composites was grade CFP-7-50 carbon fibre (CF) in powder form made by Shenzhen Yataida High-Tech. Co. Ltd. (Shenzhen, China) and supplied by Sigma Aldrich Sdn Bhd., Petaling Jaya, Malaysia. It had an average diameter of 9 µm, lengths of between 100 µm and 300 µm, and a density and aspect ratio of 1.75 g/cm^3^ and 43, respectively. Meanwhile, for the polymer resin, a grade PA2200 polyamide (PA) powder (supplied by Sigma Aldrich Sdn. Bhd., Petaling Jaya, Malaysia) was used as a binder. It had a density of 0.45 g/cm^3^, and its tensile modulus and tensile strength were 1620 MPa and 48 MPa, respectively [16]. The compound materials were premixed into two sets, 20 wt.% of CF and 40 wt.% of CF, using a mechanical mixer (IKA RW20 WERK digital, Selangor, Malaysia) at a rotational speed of 300 rpm for four minutes to ensure homogeneous mixing [13,22]. The compounded PACF was then prepared for the rheology analysis (Shimadzu CFT-500D, Selangor, Malaysia) using cylinder lengths of 10 mm and an orifice diameter of 1 mm. The rheological parameters were varied from 210 °C to 250 °C, while the load cell was set at 40 N. The prepared feedstock was then fabricated in filament form using a 3Devo filament maker at a temperature of 190 °C, an extruder speed of 5 rpm and a filament diameter of 1.75 mm as shown in Figure 1(a-1),(a-2). However, the printing temperature, printing speed and layer height were varied specifically for the 20 wt.% PACF materials, as recorded in Table 1. The printer model Ultimaker S3 was used in this study, and a schematic diagram of the machine can be found in a prior study [23]. All of these varied parameters listed were chosen in accordance with the Taguchi method and an L27 array was used, as reported in previous studies [24,25]. The printing temperature was set to range from 210 °C to 250 °C, in accordance with a previous study in which a temperature of 250 °C was used for this purpose [16]. Meanwhile, the bed temperature was set at 110 °C, the printing speed was set to 80 mm/s, and the layer height was set to 0.1 mm [16]. Existing research has indicated that the selection of printing parameters is subject to the material compositions used. Based on these findings, the prepared PACF filaments were later printed in various shapes according to the ASTM standard and the fused deposited modelling technique was used with the Ultimate S3 machine, which was set up for weights of 20 wt.% and 40 wt.%. The printing temperature for both compositions was set at 250 °C with the temperature bed, print speed and layer height set at 110 °C, 80 mm/s and 0.1 mm, respectively. 

### 2.2. Characterisation of PACF Composites

Since the filament fabrication process is crucial, the thermal behaviour, dynamic mechanical properties and viscoelasticity were determined with a thermogravimetric analysis (TGA, Quantum Skynet, Negeri Sembilan, Malaysia); differential scanning calorimetry (DSC) using a Mettler Toledo machine (Quantum Skynet, Negeri Sembilan, Malaysia); and a dynamic mechanical analyser (DMA) using Q800 (TA Instruments, New Castle, DE, USA). The temperature range was set to between 30 °C and 900 °C, with 20 °C/min increases in the temperature rate in a nitrogen environment, according to ASTM E1131-98. Meanwhile, the DMA analysis was conducted at temperatures ranging from room temperature to 150 °C, with the heating rate increasing by 1 °C/min at a frequency of 1.0 Hz, in accordance with ASTM D4092. The mechanical performance of the printed samples was determined by a contact angle analysis using a LAUDA Surface Analyzer. The analysis was performed by releasing water droplets onto the solid sample surfaces, in accordance with ASTM D7334-19. When conducting the impact test (ASTM D256-19), the samples were analysed using the Izod Pendulum Impact Test 50 J as shown in Figure 1b. The ten tests performed on each sample set (i.e., 20% infill density for 20 wt.%, 100% infill density for 20 wt.%, and 100% infill density for 40 wt.%) were conducted at room temperature. Meanwhile, to determine the shear properties of the PACF composites, the deformation resistance of a material when a force was applied parallel to its surface was analysed using the lap shear strength test (ASTM D3163-01), with the overlapping length being 25.4 mm as shown in Figure 1c. The test was conducted using the Universal Testing Machine (UTM, model Instron 5567, Universiti Kebangsaan Malaysia, Selangor, Malaysia) at a crosshead displacement rate of 1.3 mm/min at room temperature with the two sample sets (i.e., the single-piece samples (30 kN) and the two-piece samples (1 kN)) and using epoxy adhesive, Loctite EA E-20HPEpoxy. The samples were cured for 24 h [16,26]. The mechanical properties of the PACF composites were later determined through tensile testing (ASTM D638-99) at a crosshead speed of 5 mm/min using a Universal Testing Machine (UTM, model Instron 5567). The densification of the PACF composites was measured following ASTM D792 using a standard level balance (Mettler Toledo, model ME-T Analytical Balance) with 0.998 g/cm^3^ of distilled water. The aim was to determine the ability of the composite properties to change after the fabrication process. Meanwhile, the interfacial adhesion and bonding within the structural PACF composites, which are important for determining a material’s overall strength and durability, were physically analysed using a scanning electron microscope (SEM, model Quanta FEI, Quanta 400F, Quantum Skynet, Negeri Sembilan, Malaysia), having initially been coated with gold coating using a sputter coater (model Polaron Quorum Q150R) [22]. For the samples, 20 kV of accelerating voltage was used to optimise the imaging conditions, because lower accelerating voltages reveal more sensitive surface details, including the topography and morphological characteristics of the samples [27]. Furthermore, the operation took place at a low pressure and a high vacuum mode (1.3 × 10^−1^ to 1.3 × 10^−4^ Pa) to prevent the electron beam from interacting with air molecules, which can reduce the micrograph image resolution.

## 3. Effects of Filament Performance on Carbon Fibre Addition

**Figure 2:**
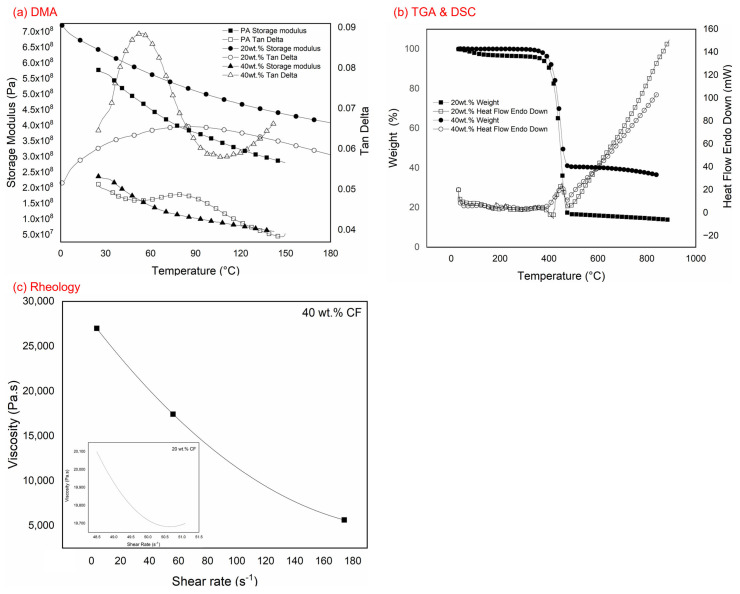
Analysis of the (**a**) dynamic mechanical analyser (DMA); and the (**b**) thermogravimetric analysis (TGA) and differential scanning calorimetry (DSC) for polyamide reinforced carbon fibre (PACF) at 20 wt.% CF and 40 wt.% CF; (**c**) Rheological analysis of both 20 wt.% and 40 wt.% CF.

**Figure 3:**
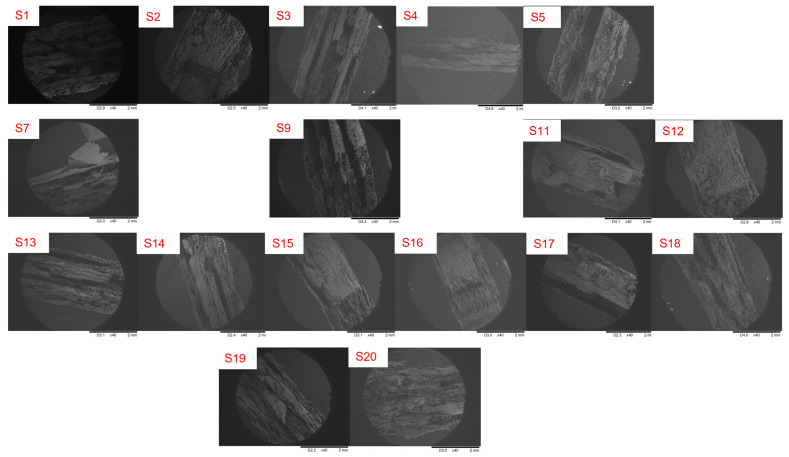
Micrograph cross-sectional images of the PACF composites with different printing parameters (refer to Table 1).

The dynamic mechanical analysis (DMA), shown in Figure 2a, indicated that the elastic modulus of a filament further affects the strength of the printed PACF samples. The filaments with 20 wt.% carbon fibre (CF) demonstrated higher storage modulus values ranging from 450 MPa to 700 MPa, whilst a 70% drop was recorded for the 40 wt.% CF, with the lowest value being 50 MPa. However, the storage modulus of pure polyamide (PA) was recorded as being 50% higher than that for 40 wt.% CF, leading to the excellent adhesion of pure PA when printed layer-by-layer [16]. This is because pure PA is less prone to warping, and no thermal mismatches occurred during printing [28]. In contrast, 40 wt.% CF exhibited the lowest storage modulus due to fibre pull-out during the extrusion (filament making) process [29]. The explanation for this can be found in previous research, which indicated that poor surface properties lead to poor bonding within the fibre matrix and a subsequent deterioration in strength [30]. Hence, having single orientations and alignment covered by the polymer matrix could be an alternative to consider further. The mechanical properties obtained through the DMA analysis were then compared in conjunction with the thermogravimetric analysis findings to determine the thermal behaviour of the polyamide reinforced carbon fibre (PACF) materials. As Figure 2b clearly indicates, 40 wt.% CF experienced better thermal stability due to the higher filler loading compared to the 20 wt.% CF, which acted as a catalyst for an improved thermal degradation process. The filler content was able to absorb more moisture, thus increasing the weight loss rate during the process. The 40 wt.% CF filler content impeded the heat transfer within the materials and initiated localised heating. In addition, Figure 2b shows that the first heating value was subjected to the thermal history of the PACF sample, whilst the cooling and second heating values indicated that the PACF materials reached a crystallinity fraction of ~50% [31]. In contrast, a study using expended graphite suggested that weight loss and thermal degradation were often experienced by samples containing gaps and multiple layers [32]. Meanwhile, the 40 wt.% samples behaved differently in terms of thermal stability, as they were more stable compared to the 20 wt.% samples. However, both compositions had an almost identical thermal degradation temperature of ~260 °C, which was used in the subsequent rheological analysis. Figure 2c shows that both the 20 wt.% and 40 wt.% samples could be extruded, and the initial viscosity was 10^3^ Pa.s. This similar rheological behaviour was also reported in a previous study using PLA in which the viscosity ranged from 10^3^ to 10^6^ Pa.s [33]. A study using polylactic acid (PLA) composites indicated that the temperature used in fused deposited modelling was often higher than when the rheological analysis was conducted [34]. Both PACF materials exhibited shear-thinning behaviour, as the viscosity decreased under the shear rate [35,36]. This pseudoplastic behaviour was influenced mainly by the entanglements within the polyamide chain in the capillary rheometer [14,37]. Furthermore, the microscopic images analysis indicated that, for 20 wt.% CF, there were obvious layup sequences of printed materials as they were printed at 210 °C and 230 °C, as shown in Figure 1(d-1),(d-2). Meanwhile, a less obvious printed layup is shown in Figure 1(d-3) for 250 °C. These findings clearly indicate that delamination occurred due to the poor adhesion between the printed samples at low printing temperatures [15,38]. A detailed analysis of the different printing parameters is shown in Table 1, whereby the printing parameters for 40 wt.% CF were chosen based on the 20 wt.% sample set. The data indicate that, even at the highest temperature of 250 °C, samples recorded a poorer tensile strength performance (~31 MPa) than at 230 °C and at the maximum pressure of 44 MPa with a 42% increment. Meanwhile, the samples were too fragile at 210 °C and their micrograph cross-sectional images could not be captured as the sample broke into layers (indicating delamination and warping issues). Research on acrylonitrile butadiene styrene (ABS) materials has indicated that utilising different printing temperatures and temperature beds are major contributors to warping issues, compared to the layer height [39,40]. Figure 3 shows micrograph images of the tensile strength of the samples in cross-sections with various printing parameters. As one study on polymer composite reinforced glass fibre indicated, the Young’s modulus of printed filaments was usually recorded as rising, except when the composition increased to 40 wt.% [6,41]. Meanwhile, the ANOVA indicated that the printing temperature had the most significant effect on the mechanical performance of the PACF composite, with a p-value of 0.04 recorded.

**Table 1:** Tensile strength results at different printing temperatures, printing speeds and layer heights for 20 wt.% polyamide reinforced carbon fibre. The parameters altered in the printing process were the printing temperature, printing speed and layer height.

Sample Name	Printing Temperature (°C)	Printing Speed (mm/s)	Layer Height (cm)	Tensile Strength (MPa)
S1	230	50	0.4	35.85
S2	250	30	0.1	31.13
S3	230	50	0.2	37.51
S4	210	70	0.3	16.28
S5	230	16	0.2	33.52
S6	210	30	0.1	26.54
S7	230	50	0.2	40.30
S8	196	50	0.2	22.99
S9	210	30	0.3	28.08
S10	250	30	0.3	25.76
S11	230	50	0.1	31.56
S12	250	70	0.1	32.50
S13	230	50	0.2	33.15
S14	230	50	0.2	37.99
S15	210	70	0.1	36.56
S16	230	83	0.2	29.38
S17	263	50	0.2	35.47
S18	250	70	0.3	41.98
S19	230	50	0.2	25.47
S20	230	50	0.2	44.17

## 4. Mechanical Performance of Printed PACF Composites

**Figure 4:**
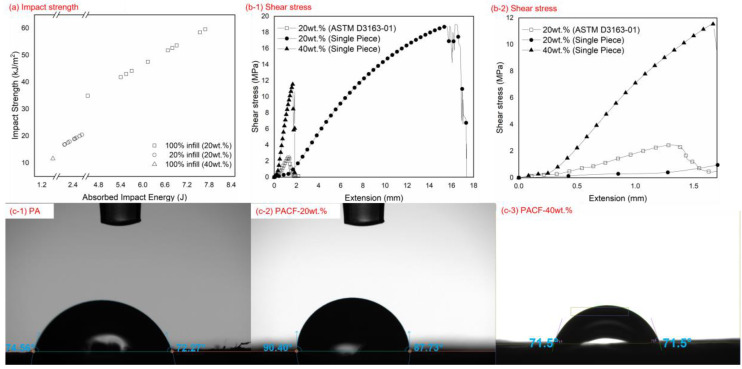
Mechanical performance of the 20 wt.% and 40 wt.% samples, including (**a**) the impact strength analysis, (**b-1**) the shear stress of single-piece samples in accordance with ASTM D3163-01, (**b-2**) an enlarged scale of shear stress in (**b-1**); a contact angle analysis of (**c-1**) pure PA, (**c-2**) 20 wt.% CF, and (**c-3**) 40wt.% CF.

The results presented in Figure 4a demonstrate the impact strengths of different infill densities, whereby the 20 wt.% CF samples with 100% infill density exhibited greater impact strengths than those with a 20% infill density. Varying the infill density between the layers provided different degrees of structural support, which reflected the overall mechanical properties of the printed PACF. Previous studies have reported that a greater impact strength can be attributed to higher infill densities, as more materials and support allow more impact energy to be absorbed [42,43]. Having 100% infill densities may seem promising as more materials are used, yet, as Figure 4a illustrates, the lower CF composition of 20 wt.% showed an increment of 500% (60 kJ/m^2^), compared to the higher CF composition of 40 wt.% (10 kJ/m^2^). This is because the printed samples started to experience delamination and warping issues with higher CF compositions due to the cohesion and poor adhesion between the layup sequences [15,44]. Meanwhile, a study using printed PLA suggested that even minor adhesion between adjacent layers can lead to subsequent full splitting of the samples, hence destroying their rigidity [45]. This finding could also be attributed to the lower shear strength shown by the 40 wt.% CF, for which a maximum value of 12 MPa was recorded, as shown in Figure 4(b-1). In contrast, for the 20 wt.% CF, the shear strength improved by 60% (19 MPa) when prepared in single-piece samples. These outcomes clearly indicate that the 20 wt.% samples had strong interlayers and excellent adhesion bonding compared to the 40 wt.% samples; these results correspond to findings obtained when polyamide-6 composites were used [46]. Meanwhile, Figure 4(b-2), an enlargement of Figure 4(b-1), demonstrates that the 40 wt.% samples had higher shear strength values due to the effects of brittleness, as reported by other research involving printed acrylonitrile butadiene styrene (ABS) composites [47]. The results also demonstrate that the samples prepared using ASTM D3163-01 had lower shear strength values than the single-piece samples. This was attributed to the shear strength of the adhesion applied on the surface (and not the layer) of the printed PACF materials, as reported in a previous study involving epoxy–polyamide composites, where the shear strength varied between 1.5 MPa and 4.65 MPa [26]. The recorded shear test data were contradicted by the contact angle data obtained, as indicated in Figure 4(c-1)–(c-3). The data demonstrate that the 20 wt.% CF produced the highest contact angle, at 87.73° (hydrophilic) and 90.4° (hydrophobic), compared to the 40 wt.% CF, for which the angle was 71.5° on both sides (hydrophilic). Studies on polymeric composites have reported that a higher contact angle indicates lower adhesion bonding from the surface to the solid substrate [48,49]. Other researchers suggest that having a hydrophilic surface indicates that the surface roughness is also higher, allowing liquid substances to be retained [48,50]. This finding is attributed to the higher shear strength of the 20 wt.% samples that that of the 40 wt.% samples, and the results show a smoother layup interface on the 20 wt.% samples [51]. Thus, a higher contact angle indicates weaker bonding between the liquid and substrate, suggesting a greater shear strength at the layup interface [21]. 

## 5. Conclusions and Future Perspectives on Polymeric Composite Filaments

This study of different filler loadings, especially those used for making filament feeders for fused deposited modelling in 3D printing is crucial, as its enables fabricated composite materials to be better understood. The analysis clearly indicates that the 20 wt.% samples had better thermal stability than the 40 wt.% samples, although both exhibited an almost identical thermal degradation temperature of ~260 °C. In terms of the mechanical performance, the 20 wt.% samples showed a 500% improvement in their impact strength, and a doubling of their shear strength performance was recorded in the analysis. Thus, the printed samples could actually be printed under higher filler loading, but post-processing, including a drying process, is required to ensure that the adhesion bonding between the layup printed samples is on par with the low filler loading samples (20 wt.%). These findings enable further understanding of the process, especially when fabricating metal-based composite materials used in fused deposited modelling. The study revealed that, by fabricating filaments with 20 wt.% filler loading and optimising the printing parameters to ensure better adhesion between layers, the resulting samples exhibit improved mechanical properties compared to conventional filaments with the same loading. The key to achieving these results is to maximise the layup sequence and minimise breakage during printing to improve the adhesion between the layers and ultimately create stronger samples. Thus, future work should focus on the correlation between the rheology analysis (relaxation timescales) with crystallisation kinetics and the timescales involved in process physics.

## Data Availability

The data presented in this study are available on request from the corresponding author.
